# Mitochondrial-Targeted Ratiometric Fluorescent Probe to Monitor ClO^−^ Induced by Ferroptosis in Living Cells

**DOI:** 10.3389/fchem.2022.909670

**Published:** 2022-06-09

**Authors:** Beidou Feng, Kui Wang, Zhe Wang, Huiyu Niu, Ge Wang, Yuehua Chen, Hua Zhang

**Affiliations:** ^1^ School of Chemistry and Chemical Engineering, Henan Normal University, Xinxiang, China; ^2^ School of Basic Medical Sciences, Xinxiang Medical University, Xinxiang, China

**Keywords:** fluorescent probe, ClO^−^, ratio fluorescence, mitochondria, ferroptosis

## Abstract

Ferroptosis is a type of iron-dependent programmed cell death. Once such kind of death occurs, an individual cell would undergo a series of changes related to reactive oxygen species (ROS) in mitochondria. A mitochondrial-targeted ratiometric fluorescent probe (**MBI-OMe**) was developed to specifically detect ferroptosis-induced ClO^−^, whose recognition group is *p*-methoxyphenol, and the mitochondrial-targeted group is benzimidazole. The fluorescence of **MBI-OMe** was first quenched by 30 μM of Fe^3+^, and then **MBI-OMe** appeared as a ratiometric signal at 477 nm and 392 nm in response to ferroptosis-induced ClO^−^ in living cells. **MBI-OMe** was successfully used to evaluate changes in ClO^−^ induced by ferroptosis.

## Introduction

Ferroptosis is a kind of programmed cell death that mainly results from the imbalance between the generation and degradation of intracellular reactive oxygen species (ROS) caused by iron accumulation (Chen et al., 2021; Cheshchevik et al., 2021; Dietrich and Hofmann, 2021). Mitochondria, the centers of energy metabolism in cells, are important sites for ROS generation (Yin et al., 2011; Li et al., 2019). Ferroptosis is frequently accompanied by the accumulation of ROS in mitochondria and is involved in many other pathophysiological processes (Gao et al., 2019; Floros et al., 2021). For example, iron homeostasis plays an important role in the nervous system, and the occurrence of ferroptosis induces neurodegenerative diseases, causing the degradation of mitochondrial activity (Wu et al., 1999; Torti et al., 2013; Zhou et al., 2022). In theory, real-time monitoring of ferroptosis could provide us with necessary information for disease diagnosis. Therefore, it is crucial to develop a selective and sensitive assay for ferroptosis-induced ROS at the solution and cellular levels, which is extremely helpful to understand the physiological and pathological roles of ferroptosis for the living organisms.

Hypochlorous acid, a kind of ROS, usually exists in the form—ClO^−^ in physiological pH (Li et al., 2012; Ren et al., 2019; Zhang et al., 2020a). Due to its oxidative properties, ClO^−^ is used in the immune defense system and plays an important role in a variety of physiological and pathological processes. Iron-dependent ferroptosis leads to the accumulation of ClO^−^ concentrations which can reach the values of 20–400 μM, leading to oxidative damage to mitochondria (Tang et al., 2021; Yan et al., 2021). Fluorescent probes are becoming more and more popular in the detection of biological objects, such as ClO^−^, because of their simplicity, high temporal and spatial resolution, and real-time and non-destructive biological imaging. To date, fluorescent probes based on the detection of ClO^−^ targeting different organelles have been designed (Wang et al., 2019; Li et al., 2020; Zhang et al., 2020b). In addition, a number of fluorescent probes have been prepared that specifically monitor ClO^−^ changes in different disease models, such as sepsis, pneumonia, and cancer (Zhang et al., 2018; Long et al., 2020; Wang et al., 2021; Yang et al., 2021). Although these probes can achieve specific, fast, and sensitive responses to ClO^−^, these probes cannot be used to detect ClO^−^ content changes in suborganelles during ferroptosis. This is mainly because the concentration of ClO^−^ produced by iron death is larger than that in normal cells, so it is impossible to determine whether ferroptosis has occurred. Therefore, it is necessary to develop related reactive oxygen species probes to selectively detect ClO^−^ and perform fluorescence imaging during ferroptosis.

With this in mind, we developed a mitochondrial-targeted fluorescent probe (**MBI-OMe**) for monitoring the changing behavior of ClO^−^ that was induced by ferroptosis in this work. **MBI-OMe** was synthesized by linking *p*-methoxyphenol with a fluorophore benzimidazole via a conjugated vinyl bond. *p*-Methoxyphenol can enhance the fluorescence intensity of the probe and act as a selective recognition site for ClO^−^ (Hu et al., 2014). **MBI-OMe** could coordinate with iron ions to quench fluorescence. When encountering ClO^−^ induced by ferroptosis, **MBI-OMe** could produce a ratiometric fluorescence signal. More importantly, **MBI-OMe** with a high fidelity signal can be used to monitor ClO^−^ production that accompanies ferroptosis, suggesting that mitochondrial ClO^−^ levels may play an important role in ferroptotic cancer cells. Therefore, **MBI-OMe** is considered to be instructive to study the detection of ClO^−^ in ferroptosis.

## Materials and Methods

### Instruments and Reagents

All chemicals and solvents used in synthesis are commercially purchased, and no further purification is performed unless otherwise stated. The structures were characterized by AVANCE III HD 600MHZ (600 MHz ^1^H, 151 MHz ^13^C) NMR spectroscopy. The solvent was DMSO (TMS as an internal standard). An ultrahigh resolution electrospray time-of-flight mass spectrometry system was used to determine the molecular weight of compounds. Fluorescence spectra were determined using the HORIBA FluoroMax-Plus spectrophotometer. The UV spectrum was determined by the Cintra 2020 spectrophotometer of GBC. Fluorescence images were collected using an Olympus FV1200 confocal laser fluorescence microscope.

### Synthesis of Probe MBI-OMe

Here, 2-methylbenzimidazole (1.5 mmol, 200.0 mg) and 2-hydroxy-5-methoxybenzaldehyde (1.8 mmol, 276.3 mg) were added to a mixed solution of acetic acid (6.0 ml) and acetic anhydride (12.0 ml). The mixture was obtained after reacting at 120°C for 6 h. The solution was cooled with water; 5 ml of concentrated hydrochloric acid was added overnight, and a solid was obtained by filtration. The yellow solid MBI-OMe was obtained by column chromatography. The yield of the probe MBI-OMe was 64%. ^1^H NMR (600 MHz, DMSO-d6) δ 12.76 (s, 1H), 9.66 (s, 1H), 7.86 (d, *J* = 16.6 Hz, 1H), 7.52 (dd, *J* = 5.9, 3.2 Hz, 2H), 7.27 (d, *J* = 16.6 Hz, 1H), 7.20–7.10 (m, 3H), 6.85 (d, *J* = 8.8 Hz, 1H), 6.80 (dd, *J* = 8.8, 2.9 Hz, 1H), and 3.75 (s, 3H). ^13^C NMR (151 MHz, DMSO-d6) δ 152.81, 151.99, 150.39, 130.86, 123.29, 122.49, 117.55, 117.34, 116.86, 111.74, and 55.91. High-resolution mass spectrometry (HRMS) m/z calcd for C_16_H_14_N_2_O_2_ (M + H)^+^: 267.1128; found 267.1156.

### Cell Imaging

HL-7702 cells and HepG 2 cells were cultured in Dulbecco’s modified Eagle’s medium (Corning) containing 10% fetal bovine serum (Sigma Aldrich) and 1% penicillin/streptomycin (Corning). Cell culture conditions were 37°C and 5% CO_2_.

For the imaging experiment of exogenous ClO^−^, HepG 2 cells were incubated with ClO^−^ solution of different concentrations for 30 min, and then the probe **MBI-OMe** was added for further incubation for 30 min. In the endogenous ClO^−^ imaging experiment, lipopolysaccharides (LPSs) were added to HepG 2 cells and incubated for 12 h; phorbol-12-myristate-13-acetate (PMA) was added for further incubation for 90 min, and then the probe was added for 30 min for imaging. The cells treated with LPS and PMA were incubated with N-acetylcysteine (NAC) and 4-azidobenzohydrazide (ABH) for 1 h and then incubated with **MBI-OMe** for 30 min for imaging. The HepG 2 cells were incubated with erastin for 6 h to construct the ferroptosis cell model and then incubated with the probe for 30 min for imaging. Deferoxamine was added and incubated for 6 h, and **MBI-OMe** was added and incubated for 30 min before imaging.

## Results and Discussions

### Design and Synthesis of MBI-OMe

In order to monitor the changing behavior of ClO^−^ during ferroptosis, a ClO^−^ responsive fluorescent probe (**MBI-OMe**) was designed. In molecular design, the *p*-methoxyphenol group was selected as the recognition site because it can produce benzoquinone through ClO^−^ oxidation. In order to realize the detection of ClO^−^ in the process of ferroptosis, imidazole and hydroxyl groups, which can complex with Fe^3+^, were introduced into the probe as the turn-on signal for the detection of ferroptosis. To form the typical intramolecular charge transfer (ICT) molecular system, **MBI-OMe** was synthesized by connecting *p*-methoxyphenol as the electron-donor group, benzimidazole compound as the electron-acceptor group, and vinyl as the conjugated bridge. When the probe is complexed with Fe^3+^, the ICT effect of the probe is weakened, and the fluorescence is quenched. When the system encounters ClO^−^, the complex system is destroyed and the fluorescence is recovered. As the ClO^−^ concentration increased, methoxyphenol was oxidized to benzoquinone, thereby exhibiting a proportional fluorescence signal.

**SCHEME 1:**
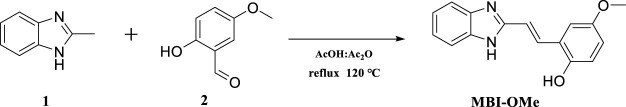
Synthesis of the fluorescent probe MBI-OMe.

As shown in Scheme 1, the synthetic route of **MBI-OMe** is simple, and **MBI-OMe** is obtained by the Knoevenagel condensation reaction of 2-methylbenzimidazole (compound 1) and 2-hydroxy-5-methoxy-benzaldehyde (compound 2) directly. The chemical structure of **MBI-OMe** was characterized by ^1^H NMR, ^13^C NMR, and ESI-TOF-MS.

## Spectral Properties

**FIGURE 1:**
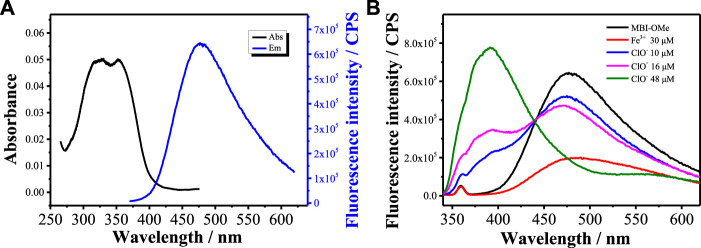
**(A)** Absorption and emission spectra of MBI-OMe (2.0 μM) in PBS buffer (pH = 7.4). **(B)** Emission spectra of MBI-OMe in response to Fe^3+^ (30 μM) and ClO^−^ (10, 16, and 48 μM).

The optical properties of the **MBI-OMe** (2.0 μM) were investigated by UV-vis absorption and fluorescence spectroscopy in PBS buffer (pH = 7.4). As shown in Figure 1A, **MBI-OMe** showed bright fluorescence at 477 nm that was excited by the one-photon light source (λ_ex_^one-photon^ = 325 nm, Φ = 0.28) and the two-photon light source (λ_ex_^two-photon^ = 750 nm, Supplementary Figure S1). When **MBI-OMe** encountered 30 μM of Fe^3+^ in PBS buffer (pH = 7.4), the fluorescence decreased to 1.5×10^5^ at 477 nm (Φ = 0.015) within 1 min (Supplementary Figure S2). Also, the dosage of Fe^3+^ for the **MBI-OMe** is 30 μM, which is far greater than the endogenous amount of Fe^3+^ in living organisms. Therefore, the Fe^3+^ concentration increased to 30 μM when ferroptosis occurred (Hentze et al., 2010). Due to the complexation of a large amount of Fe^3+^ with **MBI-OMe**, resulting in the change of the intramolecular conjugated system, the fluorescence intensity is quenched (Zhao et al., 2016). After that, by adding ClO^−^ into the system, the fluorescence of **MBI-OMe** at 477 nm (Φ = 0.25) was recovered within 1 min (Supplementary Figure S2). When ClO^−^ was continually added into the system, fluorescence strength at 477 nm decreased and was accompanied by another increase at a new emission peak (392 nm, Figure 1B).

**FIGURE 2:**
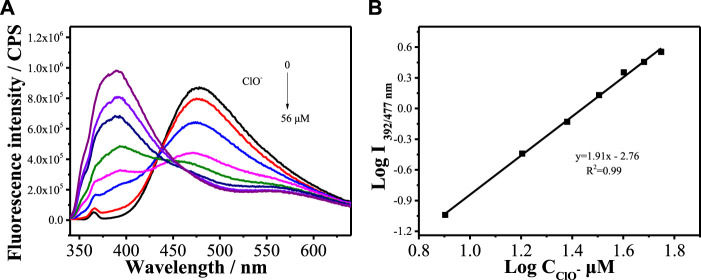
**(A)** Fluorescence spectra of MBI-OMe (2.0 µM) in PBS buffer (pH = 7.4) containing different concentrations of ClO^−^ (0–56 μM). **(B)** Linear relationship between MBI-OMe fluorescence intensity and ClO^−^ concentration.

Figure 2A shows the fluorescence spectra of **MBI-OMe** in PBS buffer (pH = 7.4) containing different concentrations of ClO^−^ (0–56 μM) at an excitation wavelength of 325 nm. With the increase of ClO^−^ concentration, the fluorescence at 477 nm gradually weakened, a new fluorescence emission peak appeared at 392 nm, and the fluorescence intensity gradually increased with the increase of ClO^−^ concentration. The emission peak at 392 nm increased 40-fold after the addition of 56 μM ClO^−^. As can be seen in Figure 2B, the fluorescence intensity has a good linear relationship with the concentration of ClO^−^ in the range of 8–56 μM. The detection limit of **MBI-OMe** for ClO^−^ is 1.2 μM. The results showed that the probe could interact with ClO^−^ with good sensitivity.

To evaluate the selectivity of **MBI-OMe** for ClO^−^, the fluorescence responses to various analytes, including biologically reactive oxygen species, amino acids, and cationic and anionic species, were investigated in PBS buffer (pH = 7.4). As shown in Supplementary Figure S4, there were no significant changes in other bioactive species, except for the ClO^−^-induced spectra change. Meanwhile, in order to prove that **MBI-OMe** can have good photostability *in vivo*, the fluorescence intensity of the PBS solution containing **MBI-OMe** after illumination at different times was recorded. Supplementary Figure S5 shows that the fluorescence intensity decreases slightly with the increase of time, but the intensity remains above 80% after 5 h, which indicates that **MBI-OMe** has good photostability. The results showed that **MBI-OMe** has a good fluorescence response to ClO^−^, which is very suitable for biological applications.

## Sensing Mechanism

**SCHEME 2:**
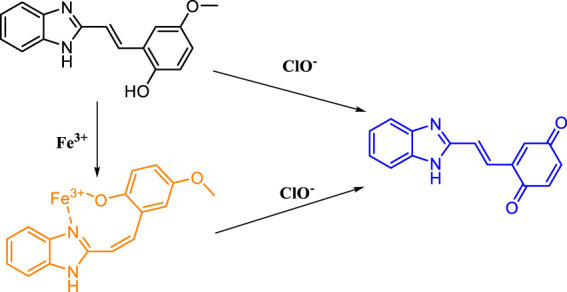
Molecular structure of MBI-OMe and the proposed sensing mechanism.

Based on the aforementioned spectroscopic evidence, a possible mechanism for the detection of ClO^−^ and Fe^3+^ by **MBI-OMe** was proposed (Scheme 2). The sensing mechanism was detected by adding ClO^−^ and Fe^3+^ to the **MBI-OMe** probe solution. The reaction solution was detected by high-resolution mass spectrometry (HRMS). When Fe^3+^ (30 μM) was added into **MBI-OMe** (2.0 μM), the mass spectrum showed a peak at 267.1126 M/z [M + H^+^]. This indicated that the probe only complexed with Fe^3+^ without changing the molecular structure. When ClO^−^ (20 μM) was added into it, there were two peaks showed by the mass spectrum at 267.1121 M/z [M + H^+^] and 249.0663 M/z [M-H^+^]. This indicated that with the addition of ClO^−^, the phenolic hydroxyl and methoxy groups in the probe were oxidized to benzoquinone. In addition, mass spectrometry analysis showed 251.0790 M/z [M + H^+^] after adding amounts of ClO^−^ to the probe-containing solution (Supplementary Figure S6). It was further proved that *p*-methoxyphenol was oxidized to benzoquinone in the probe, and a proportional fluorescence signal was generated.

### Chemical Stability and Biological Toxicity

**FIGURE 3:**
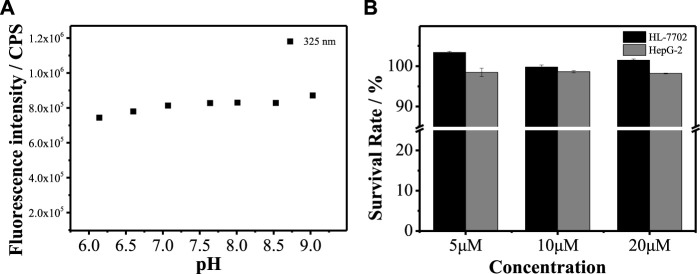
**(A)** Fluorescence signal changes of MBI-OMe (2.0 µM) at different pH (6.0–9.0). **(B)** Cytotoxicity of MBI-OMe at different concentrations (5, 10, and 20 µM).

To verify whether **MBI-OMe** can be used for bioimaging detection, the chemical stability of **MBI-OMe** in the physiological pH range was first investigated. As shown in Figure 3A, it can be seen that the fluorescence intensity of **MBI-OMe** remains almost unchanged with the change of pH 6.0–9.0. This indicates that **MBI-OMe** has good chemical stability and can be used for testing in a physiological environment. To further illustrate the feasibility of the probe for the imaging detection in living cells, the cytotoxicity was measured by MTT assay in HL-7702 cells and HepG 2 cells. Figure 3B showed that the cell viability was maintained above 95% under incubation by different concentrations of **MBI-OMe**, showing good cell viability. **MBI-OMe** proved to be a potential tool for detecting and imaging in living cells.

### Ratiometric Fluorescence Detection and Imaging of ClO^−^

**FIGURE 4:**
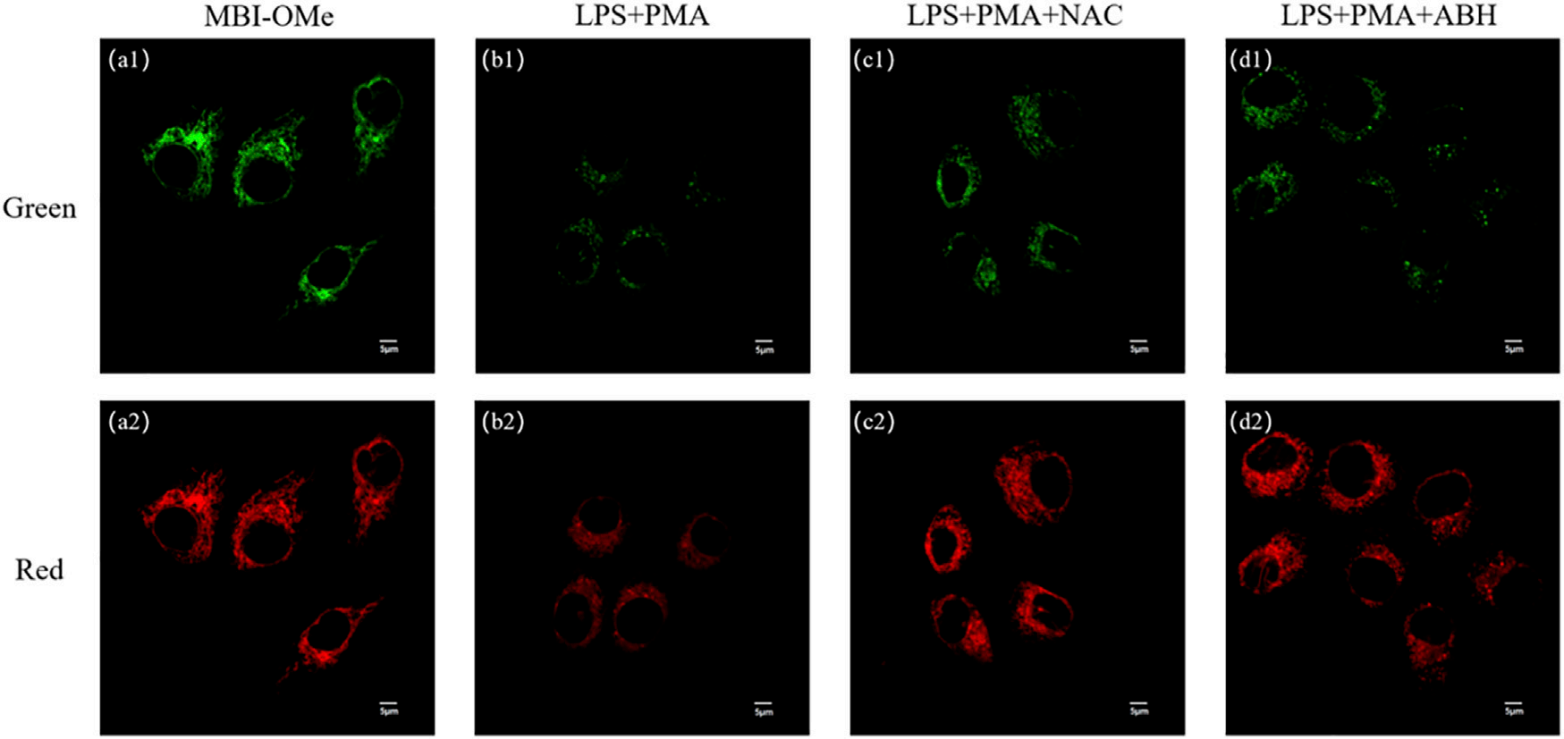
**(A)** Imaging of MBI-OMe in HepG 2 cells. **(B)** Cell imaging of MBI-OMe performed after LPS (1.0 μg/ml, 12 h) and PMA (10 mg/ml, 90 min) were added to the cells. **(C)** Cell imaging of MBI-OMe after incubation with NAC (2.0 mM, 1 h) under the condition of **(B)**. **(D)** Cell imaging of MBI-OMe after incubation with ABH (200 μM, 1 h) under the condition of **(B)**. Green: 415–450 nm; Red: 460–560 nm; MBI-OMe: 30 µM.

We first explored whether this probe could be used for imaging (Supplementary Figure S7A) in living cells. When HepG 2 cells were incubated with **MBI-OMe**, there appeared a distinct fluorescence and presented good regional characteristics. The colocalization experiments demonstrated that **MBI-OMe** was able to localize in the mitochondria (Supplementary Figure S7). In addition, two-photon imaging in cells showed that the probe had a good two-photon effect (Supplementary Figure S8). These results suggested that **MBI-OMe** can be used to image intracellular mitochondria. By adding ClO^−^ to cells, it was demonstrated that the probe could detect and image ClO^−^ in cells (Supplementary Figure S9). Subsequently, the endogenous ClO^−^ production was induced in cells by adding lipopolysaccharide and phorbol, as shown in Figures 4A,B. The fluorescence of **MBI-OMe** in both the red channel (460–560 nm) and green channel (415–450 nm) was weakened. This may be due to the fact that the excitation wavelength is not optimal (Ex = 405 nm, the best excitation wavelength is 325 nm), which leads to the decreased fluorescence intensity of the probe in the green channel. In order to verify whether endogenous ClO^−^ responded to the probe, NAC and ABH were added to cells, as shown in Figures 4C,D. When ClO^−^ was removed from cells, the probe was added for incubation, and the fluorescence intensity was basically the same as that of the probe alone. The aforementioned experiments indicate that **MBI-OMe** may be an effective tool for detecting and imaging ClO^−^ in mitochondria of ferroptosis cells.

### Changes of ClO^−^ During Cell Ferroptosis

**FIGURE 5:**
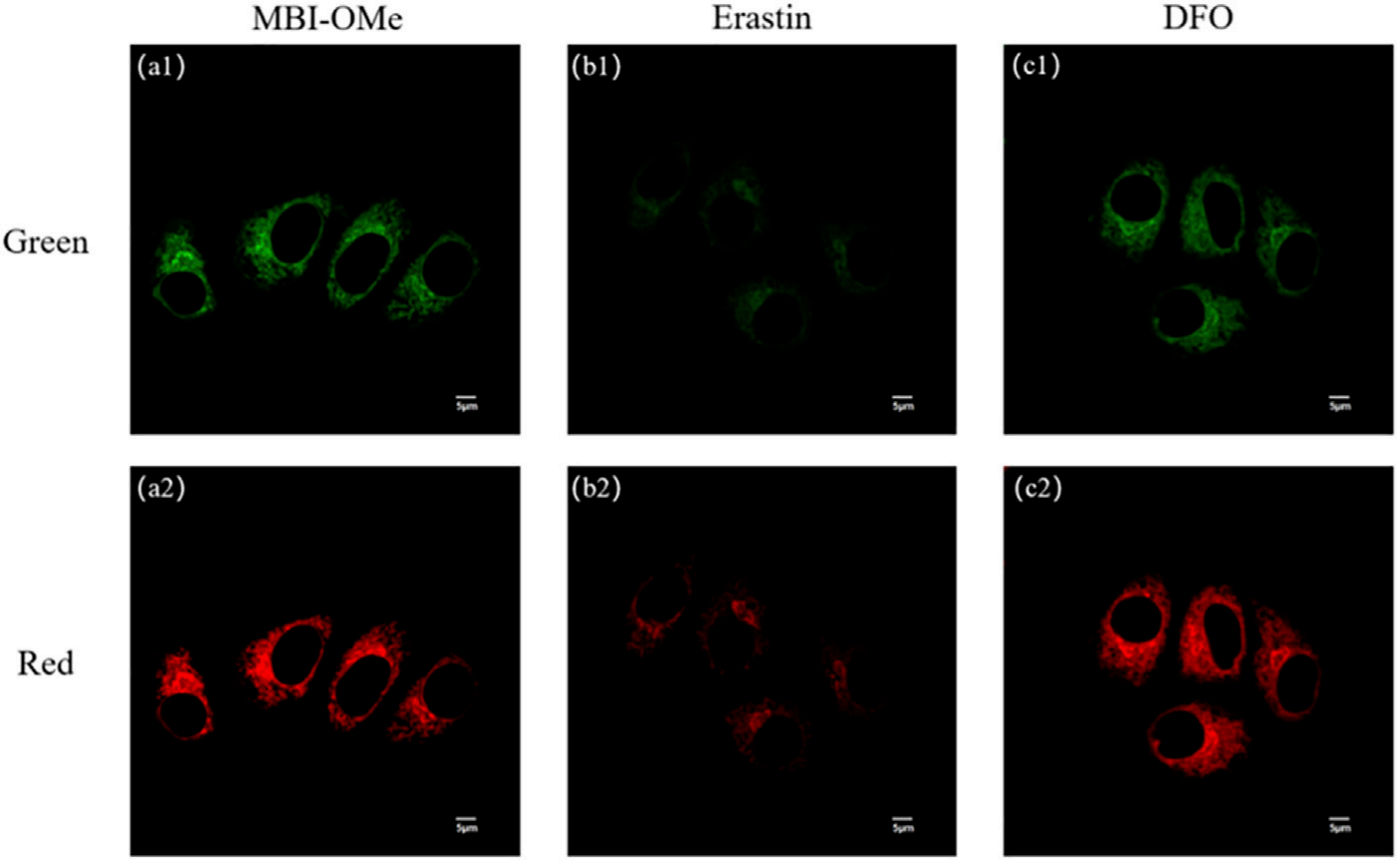
**(A)** Imaging of MBI-OMe in HepG 2 cells. **(B)** Cell imaging of MBI-OMe performed after erastin (10 μM, 6 h) was added to the cells. **(C)** Cell imaging of MBI-OMe after incubation with DFO (100 μM, 6 h) under the condition of **(B)**. Green: 415–450 nm; Red: 460–560 nm; MBI-OMe: 30 µM.

Ferroptosis is a novel mode of cell death distinct from apoptosis, necrosis, and autophagy (Dixon et al., 2012; Li, 2020). The most characteristic feature of ferroptosis is the need for iron, and some iron chelators can inhibit this process. Studies have shown that the production of ROS is observed during ferroptosis (Li et al., 2019; Chen et al., 2020; Zhang et al., 2020a). Here, the ability of **MBI-OMe** to image ClO^−^ during ferroptosis in HepG 2 cells was further investigated by constructing a cellular ferroptosis model. As shown in Figure 5, the fluorescence intensity of the green and red channels was significantly attenuated after treatment of cells with erastin, a classic iron ion inducer (Dixon et al., 2012), for 6 h, compared with untreated HepG 2 cells. Deferoxamine (DFO, an inhibitor of ferroptosis) significantly inhibits the onset of ferroptosis (Dixon et al., 2012). When DFO was added to the treated cells and incubated, the fluorescence intensity of the probe was basically the same as that of the probe only, which was the same as that of the *in vitro* spectroscopic experiment and intracellular ClO^−^ imaging experiment. The aforementioned results suggest that the probe **MBI-OMe** can be used as an imaging sensor for ClO^−^ changes during ferroptosis in cancer cells.

## Conclusion

In summary, **MBI-OMe** based on benzimidazole was developed for selective detection of ClO^−^ in mitochondria during ferroptosis. The *p*-methoxyphenol group is used as the specific reaction site of ClO^−^, and methylbenzimidazole is used for the mitochondrial targeting. **MBI-OMe** appears as a fluorescence at 477 nm. **MBI-OMe** can form a complex with Fe^3+^, and its fluorescence also was quenched (Φ = 0.015). More importantly, **MBI-OMe** can not only complex with Fe^3+^ for fluorescence quenching but also show a good ratiometric fluorescence signal to ClO^−^ that was caused by ferroptosis stimulation. The fluorescence intensity ratio (I_392_ nm/I_477_ nm) was linearly related to the concentration of ClO^−^ with a detection limit of 1.2 μM. This can mainly be attributed to the formation of benzoquinone through the redox reaction between ClO^−^ and *p*-methoxyphenol. **MBI-OMe** has good two-photon properties, which is beneficial to the detection and imaging of ClO^−^ in ferroptosis. This work provides an effective tool for the detection of ferroptosis and its ClO^−^-related diseases. In this work, the probe was able to achieve a proportional fluorescence signal *in vitro* to detect ClO^−^. However, the excitation light wavelength is in the range of 275–400 nm in one-photon mode. Although the probe has two-photon properties, in order to broaden the application of this type of probe in biological detection and imaging, it is still necessary to improve the wavelength of the probe under single-photon excitation. Therefore, more long-wavelength fluorescent probes need to be designed for the diagnosis of biological diseases.

## Data Availability

The original contributions presented in the study are included in the article/Supplementary Material; further inquiries can be directed to the corresponding author.
