# Supplementary material for: Mitochondrial-Targeted Ratiometric Fluorescent Probe to Monitor ClO− Induced by Ferroptosis in Living Cells
Source: Front Chem. 2022 Jun 9;10:909670. doi: 10.3389/fchem.2022.909670 (PMC9218690; doi:10.3389/fchem.2022.909670)
Supplement: Supplementary file 1 [file DataSheet1.PDF]

Supplementary Material

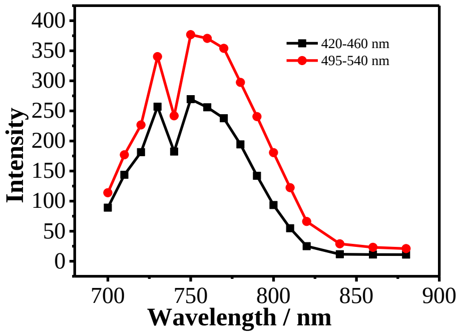

Fig. S1 Fluorescence intensity of MBI-OMe (30 μM) in cells under different two-photon excitation wavelengths

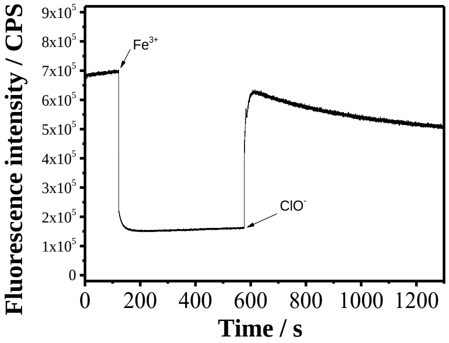

Fig. S2 Kinetic response of MBI-OMe (2.0 μM) with Fe^{3+} (30 μM) and ClO^- (10 μM) in PBS buffer (pH = 7.4)

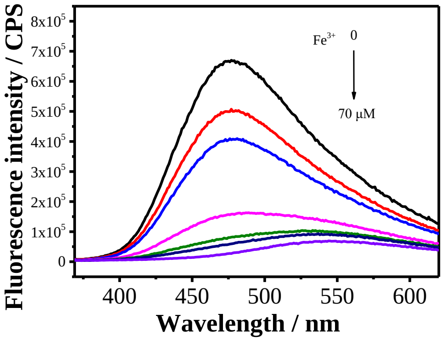

Fig. S3 Fluorescence spectral response after adding 0-70 μM of Fe^{3+} to MBI-OMe (2.0 μM) in PBS buffer (pH = 7.4)

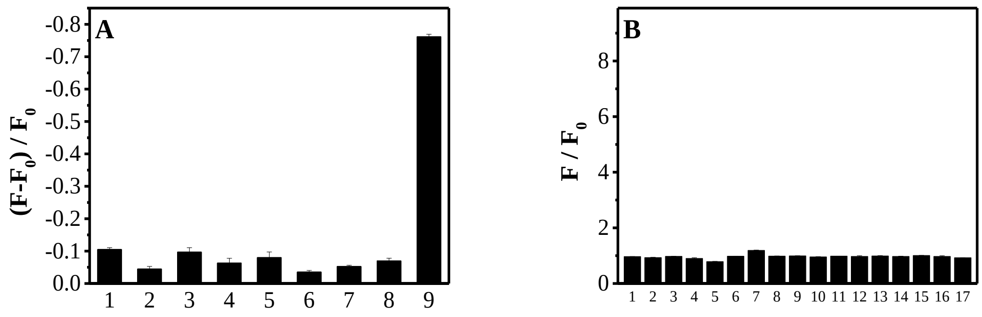

Fig. S4 Determination of MBI-OMe (2.0 μM) interference with related active small molecules in organisms. (A) Reactive oxygen species. 1 $ONOO^-$; 2 H_2O_2; 3 ·OH; 4 NO·; 5 TBO·; 6 $^1O_2$; 7 O^{2-}; 8 TBHP; 9 ClO^- (200 μM). (B) 1 $CaCl_2$; 2 $MnCl_2$; 3 $CoCl_2$; 4 $CaSO_4$; 5 $FeCl_2 \cdot 7H_2O$; 6 $MgSO_4$; 7 $Hg(NO_3)_2$; 8 $AgNO_3$; 9 lysine; 10 DL-threonine; 11 glutamine; 12 cystine; 13 glutamic acid; 14 arginine; 15 hypoxanthine; 16 L-aspartic acid; 17 Glutathione (200 μM). (F_0: the initial fluorescence intensity of probes MBI-OMe; F: the fluorescence intensity after adding interference)

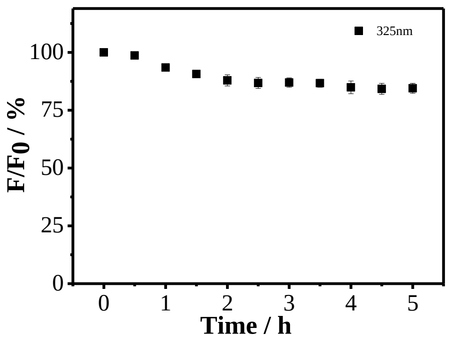

Fig. S5 Photostability test of MBI-OMe (2.0 μM) in PBS buffer (pH = 7.4). (F_0: the initial fluorescence intensity of probe MBI-OMe; F: the fluorescence intensity after illumination)

This is a provisional file, not the final typeset article

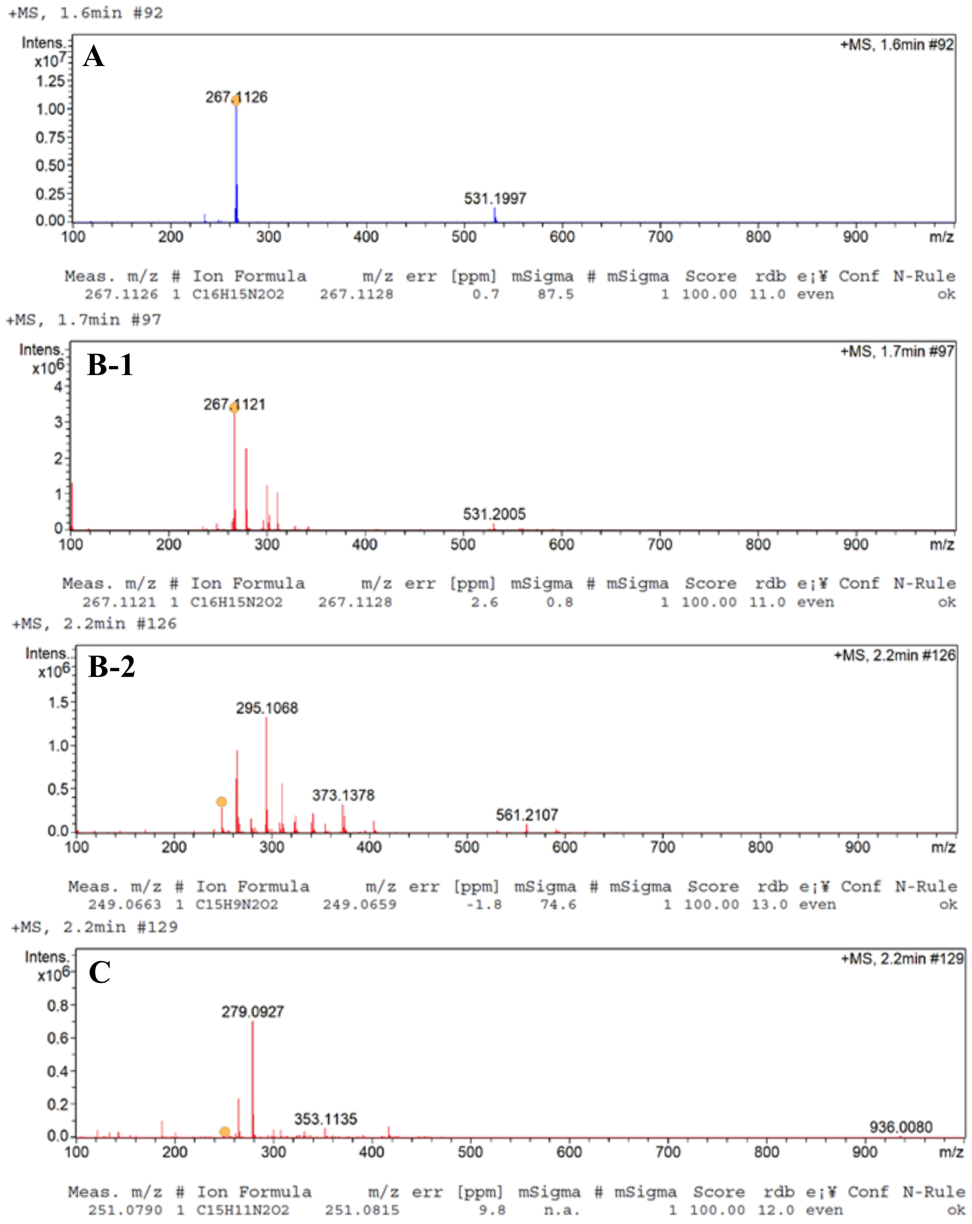

Fig. S6 The sensing mechanism was detected by adding ClO^- (1.0 mM) and Fe^{3+}(0.5 mM) to the MBI-OMe (0.2 mM) probe solution. (A) MBI-OMe+Fe^{3+}, (B) MBI-OMe+Fe^{3+}+ClO^-, (C) MBI-OMe+ClO^-

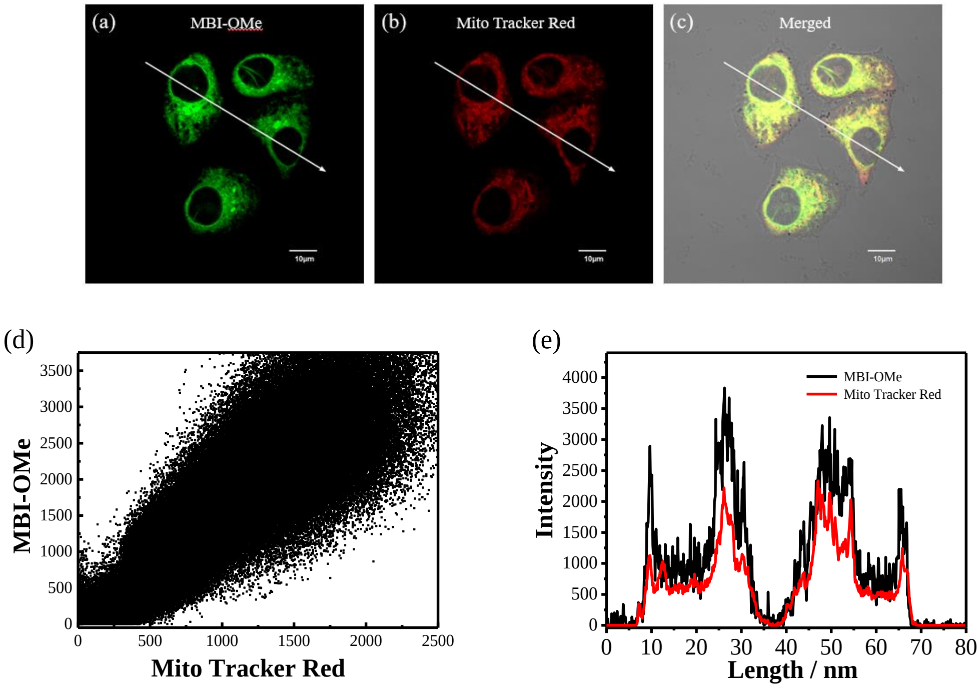

Fig. S7 Cell imaging and cell colocalization experiment. (a) Stained with MBI-OMe (15 μM). (b) Stained with Mito Tracker Red (1.0 μM). (c) Merged image of Mito Tracker Red and MBI-OMe. (d) Intensity correlation plot of stain MBI-OMe and Mito Tracker Red. (e) Intracellular coregionalization of MBI-OMe and Mito Tracker Red. The excitation wavelength of MBI-OMe is 405 nm and the fluorescence collection range is 460–510 nm; the excitation wavelength of Mito Tracker Red is 559 nm and the fluorescence collection range is 580–620 nm. The scale bar represents 10 μm

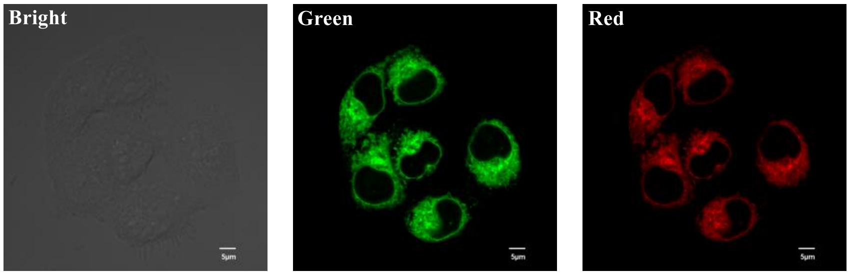

Fig. S8 Two-photon imaging of MBI-OMe in HepG 2 cells. Green: 420-460 nm; Ex: 750 nm; Red: 495-540 nm; MBI-OMe (30 μM)

This is a provisional file, not the final typeset article

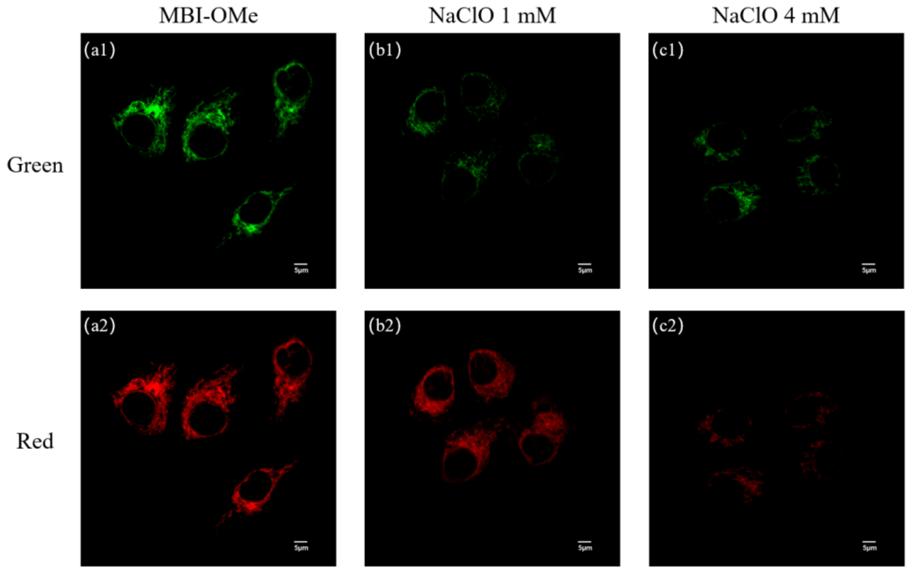

Fig. S9 a) The imaging of MBI-OMe in HepG 2 cells. b) The cell imaging of MBI-OMe after adding 1 mM ClO^- into cells. c) The cell imaging of MBI-OMe after adding 4 mM ClO^- into cells. Green: 415-450 nm; Red: 460-560 nm; MBI-OMe (30 μM)

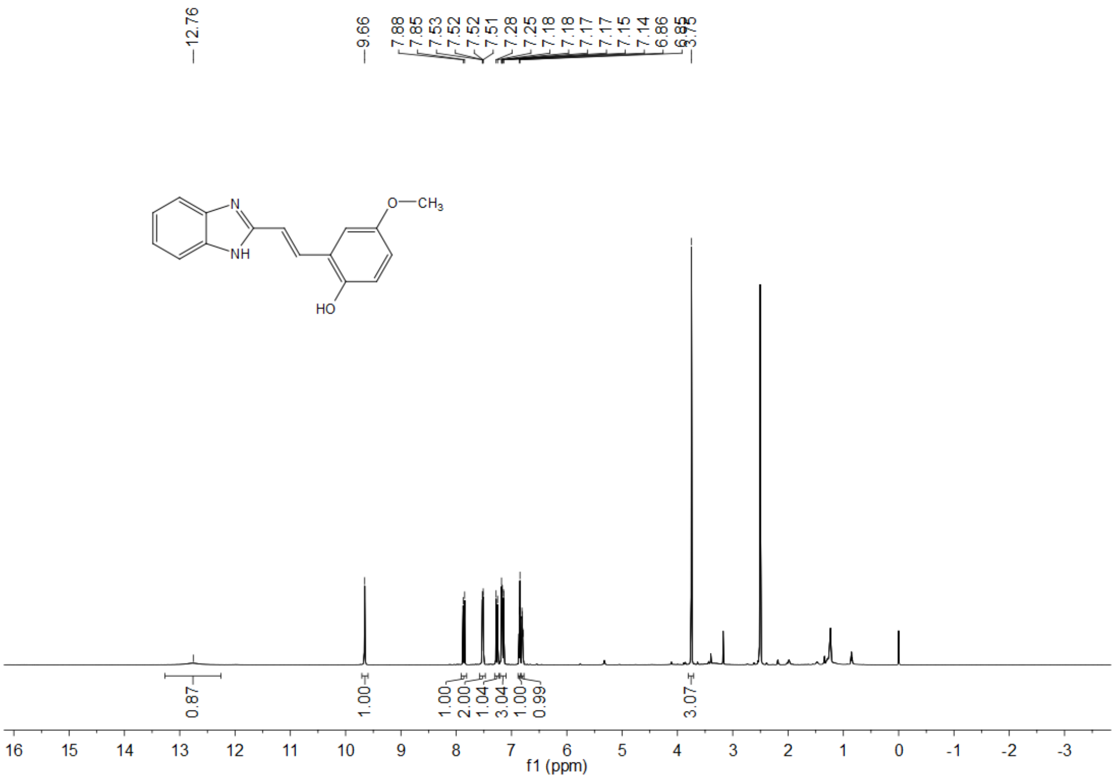

Fig. S10 ^{1}H NMR of compound MBI-OMe

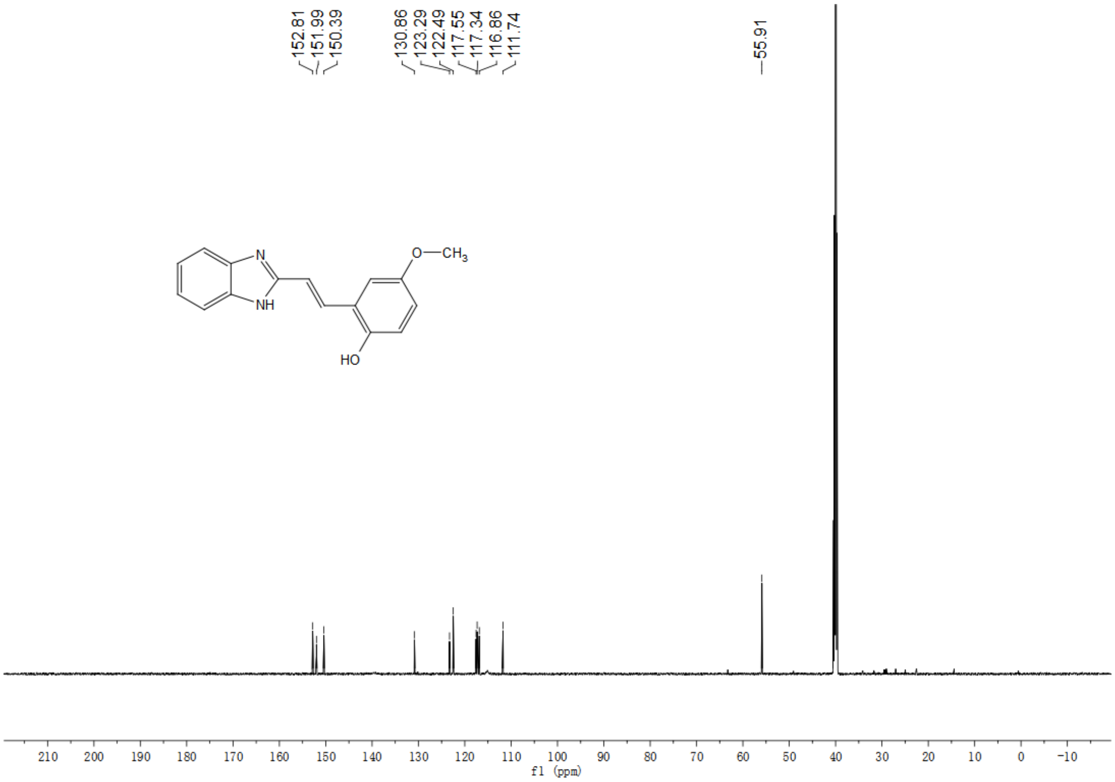

Fig. S11 ^{13}C NMR of compound MBI-OMe

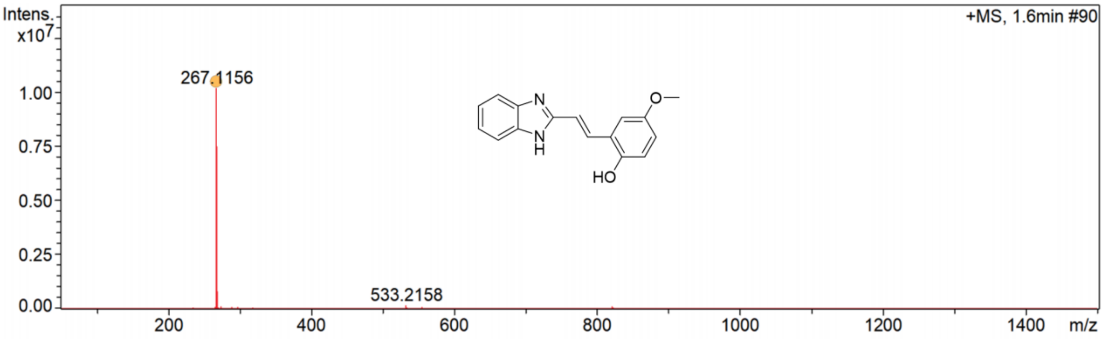

Meas. m/z	#	Ion Formula	m/z	err [ppm]	mSigma	# mSigma	Score	rdb	e¡¥ Conf	N-Rule
267.1156	1	C16H15N2O2	267.1128	-10.5	320.4	1	100.00	11.0	even	ok

Fig. S12 Mass spectroscopy of compound MBI-OMe

This is a provisional file, not the final typeset article
